# Predictors of Prolonged Hospitalization in Pediatric Burn Patients: Insights From a Rural Burn Intensive Care Unit (BICU) in Appalachia

**DOI:** 10.7759/cureus.77589

**Published:** 2025-01-17

**Authors:** Armein Rahimpour, Emily Saurborn, Nathan Fox, Gerard V Giangrosso, David Denning, Curtis W Harrison, Paul Bown, Rahman Barry

**Affiliations:** 1 General Surgery, Marshall University Joan C. Edwards School of Medicine, Huntington, USA; 2 Dermatology, Marshall University Joan C. Edwards School of Medicine, Huntington, USA; 3 Plastic and Reconstructive Surgery, Marshall University Joan C. Edwards School of Medicine, Huntington, USA

**Keywords:** burn predictors, burn source, pediatric burns, pediatric morbidity, socioeconomic factors, total body surface area (tbs), total hospital duration (thd)

## Abstract

Introduction: Burn injuries are the fifth most common cause of non-fatal injuries among children worldwide. Pediatric burn patients require complex management due to their distinct physiology compared to adults. The purpose of this study is to investigate demographic and clinical factors contributing to a prolonged total hospital duration among pediatric burn patients in a rural, Level 2 Trauma Center and Burn Intensive Care Unit (BICU) located in the Appalachian region.

Methods: Data were collected from 2017 to 2023 and included all patients 18 years and younger who were admitted to the Cabell Huntington Hospital BICU. Patient demographics were analyzed using descriptive statistics. One-way ANOVA assessed the differences between gender and length of stay (LOS) and source of burn and LOS. A chi-square test analyzed the effect of an inhalation injury on LOS. Pearson correlation was used to assess the relationship between BMI and LOS and between total ventilation days (TVD) and LOS.

Results: A total of 232 patients were included in this study, consisting of 102 (44%) females and 130 (56%) males. The average patient age was 6.9 years (SD ± 6.2). The mean LOS was 3.1 (SD ± 4.4) among this cohort. The most common sources of burn injuries within this cohort were flame (43%), scald (35%), and other (chemical/electric/radiation) comprising 22%. Flame burns were found to have a significant impact on LOS (p = 0.039), requiring longer hospital stays compared to other burn sources. Age was a significant predictor, with each additional year of age associated with an increase of 0.15 days in LOS (95% CI: 0.05 to 0.25, p < 0.001). Similarly, the total body surface area (TBSA) showed a significant positive association, with larger burn areas strongly predicting longer hospital stays (p < 0.001). The median TBSA was 5.5% (SD ± 6.7). For every 1% increase in TBSA burns, the hospital stay increased by approximately 0.13 days (95% CI: 0.05 to 0.22, p < 0.05). However, variables such as inhalation injury (p = 0.748) and BMI (p = 0.058) did not significantly predict hospital duration.

Conclusion: The results of this study demonstrated that age, burn severity, and the source of the burn are significant predictors of prolonged hospital stay in pediatric burn patients. Recognizing these key predictors of LOS will allow providers to identify high-risk patients early and initiate prompt stabilization, management, and timely referral to tertiary centers when necessary. Further, the results of this study may serve as the framework for the development of preventative efforts and regional care guidelines for rural, underserved healthcare providers.

## Introduction

Burn injuries are the fifth most common cause of non-fatal injuries among children worldwide [1]. Pediatric patients are particularly susceptible to severe injuries due to their natural curiosity and limited ability to perceive risks [2]. Burns frequently lead to emergency department visits for pediatric patients, as they often result in severe injury and pain [3]. Stabilizing the patient and initiating proper medical management early in the patient's presentation is important, as extreme burns are a significant cause of mortality worldwide [1]. Additionally, many burn patients experience severe, debilitating pain and potential disfigurement, resulting in psychosocial stress later in the recovery period [2]. Therefore, a multidisciplinary approach is crucial to ensuring a successful recovery for these patients.

Managing pediatric burn patients is particularly complex due to their distinct physiology compared to adults. Further, the lack of clear scoring guidelines for the pediatric population presents a significant challenge. Scoring systems such as Baux and ABSI (abbreviated burn severity index) are tailored to adults and often overestimate mortality rates in pediatric patients [4]. Therefore, understanding the key variables associated with poor outcomes in pediatric patients serves to guide early management. For example, younger children have a thinner epidermis compared to older children, placing them at an increased risk of deeper and more severe burns at lower temperatures [5]. Additionally, children have different body proportions than adults: a larger head relative to total body surface area (TBSA) and smaller contributions from their legs [6]. The differences in body surface area predispose pediatric patients to greater burn sizes, placing them at an increased risk for prolonged ICU stays and overall worse outcomes [7]. Despite these challenges, pediatric patients tend to have fewer secondary comorbidities to complicate management, making their hospital course primarily determined by their degree of burn [7]. Recognizing the differences between adult burns and pediatric burns is important for early stabilization and management and understanding when to refer to tertiary burn centers.

Currently, there is limited research on predictors of length of hospital stay (LOS), defined as the number of days of inpatient stay, among pediatric burn patients, notably in the Appalachian region. Appalachia is a rural region in the eastern United States extending from Pennsylvania to Alabama. With its predominantly rural geography, higher poverty rates, and dependence on industrial work, individuals residing in this region are at a heightened risk for severe burn injuries [8]. Literature suggests that rural residence, male gender, flame burns, and lower socioeconomic status are key predictors of severe outcomes [5].

The purpose of this study is to investigate demographic and clinical factors contributing to prolonged LOS among pediatric burn patients in Appalachia. This study was conducted at a Level 2 Trauma Center and Burn Intensive Care Unit (BICU) affiliated with the Joan C. Edwards School of Medicine Department of General Surgery at Cabell Huntington Hospital in Huntington, West Virginia. This study provides an analysis of factors associated with common burn injuries and those contributing to a prolonged hospital stay within the Appalachian region.

This study aims to provide insights into understanding key predictors of poor outcomes to improve initial stabilization and management of pediatric burn patients. By recognizing the factors contributing to severe pediatric burns, healthcare providers in Appalachia can provide improved early management and timely referrals to tertiary care centers when necessary. This is particularly critical in regions with limited healthcare access, where early stabilization and effective treatment plans can prevent a prolonged hospital stay and worse outcomes for these patients.

## Materials and methods

Study approval and design

The study received approval from the Marshall University Institutional Review Board (IRB no. 2063568-1). A retrospective analysis was conducted on pediatric burn patients (≤18 years old) admitted to a BICU at a rural Level 2 Trauma Center. Patient records were obtained from January 1, 2017, to January 1, 2023, and were reviewed to identify eligible cases for inclusion.

Data collection

Patient data were extracted from the hospital's electronic medical record system in collaboration with the information technology (IT) department. The initial data extraction included all patients diagnosed with burns during the study period, resulting in a sample of over 1,300 patients. After detailed chart reviews, pediatric patients aged ≤18 years were included. Patients with misdiagnoses such as Stevens-Johnson syndrome, road rash, frostbite, or those without documented burns were excluded, resulting in a final sample of 232 pediatric burn patients.

The variables analyzed in this study included demographic variables (age and gender) and clinical variables (TBSA, source of burn, inhalation injury, BMI, total ventilation days (TVD), and LOS). TBSA was assessed using the Wallace rule of nines. The source of the burn was categorized as either flame, scald, or other (chemical, electrical, or radiation). Inhalation injury was diagnosed based on the presence of carbonaceous material or soot present in the oropharynx, in addition to difficulty with oxygenation. BMI was calculated from the recorded height and weight at admission. TVD was calculated based on the number of days the patient required mechanical ventilation. The outcome variable in this study was LOS, calculated by the number of days the patient remained in the BICU.

Statistical analysis

Patient demographics were analyzed using descriptive statistics, with continuous variables expressed as means ± standard deviations (SD), while categorical variables were reported as frequencies and percentages. Statistical analyses evaluated the relationships between various predictor variables and LOS. A one-way ANOVA assessed the differences between gender and LOS and between the source of burn and LOS. A chi-square test analyzed the effect of an inhalation injury on LOS. Pearson correlation was used to assess the relationship between BMI and LOS and between TVD and LOS. However, limited TVD data limited the latter analysis.

Regression modeling

A linear regression model was used to quantify the impact of continuous variables (age, TBS) and categorical variables (burn source, inhalation injury) on LOS. Regression coefficients provided an estimate of the additional days of hospital stay associated with each predictor.

Data management and statistical tools

All data were centralized and managed using Microsoft Excel (Microsoft Corporation, Redmond, WA). Statistical analyses were conducted using Python (version 3.8) and the Statsmodels library for regression analysis. Statistical significance was defined as p < 0.05.

## Results

**Figure 1:**
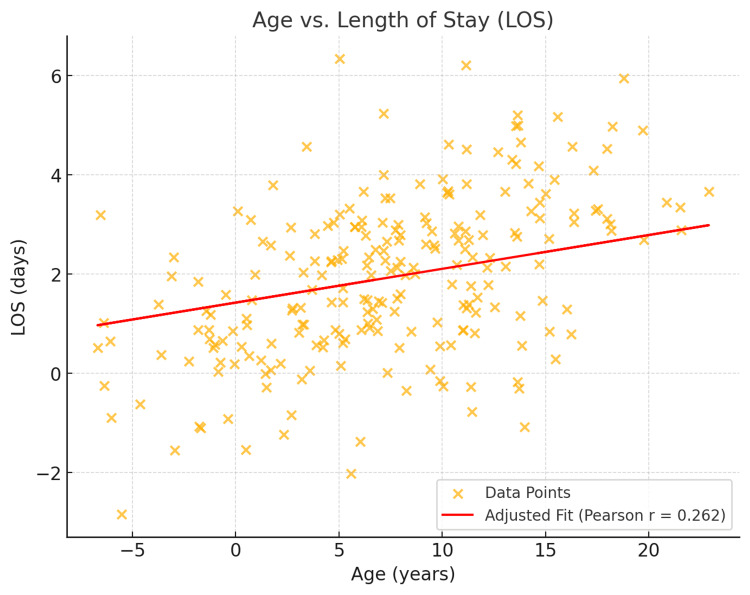
Age vs. length of stay (LOS)

**Figure 2:**
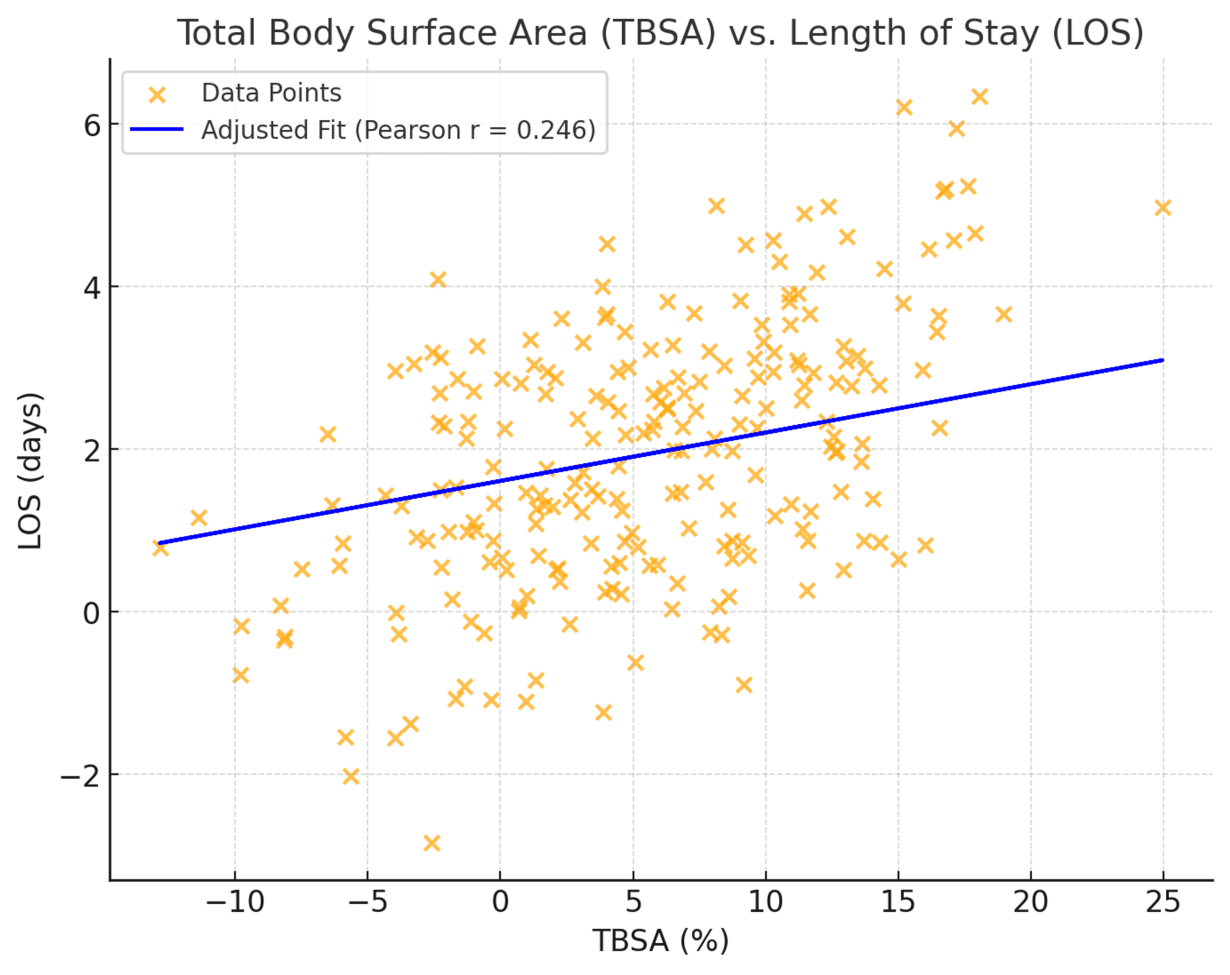
Area (TBSA) vs. length of stay (LOS) TBSA, total body surface area

A total of 232 patients were included in this study, with demographic and clinical characteristics summarized in Table 1. The average patient age in this study was 6.9 years (SD ± 6.2). Older patients (patients above the average age of the population) were found to have a significantly longer hospital stay compared to younger patients (Pearson correlation = 0.262, p < 0.001, t = 4.10). Each additional year of age is associated with an increase of 0.15 days in LOS (95% CI: 0.05 to 0.25, p < 0.001). Burn severity, measured by TBSA, averaged 5.5% (SD ± 6.7) within this cohort. A significant positive correlation was found between TBSA and LOS (Pearson correlation = 0.246, p < 0.001, t = 3.86) (Figure 1), indicating that larger burns were strongly associated with prolonged hospital stays. For every 1% increase in TBSA burns, the hospital stay increased by approximately 0.13 days (95% CI: 0.05 to 0.22, p < 0.05). Further, a weak positive correlation was found between BMI and LOS (Pearson correlation = 0.125, p = 0.058, t = 1.91) (Figure 2), although this finding did not reach statistical significance.

**Table 1:** Length of stay (LOS) in relation to age, BMI, and total body surface area (TBSA) in pediatric burn injuries Statistical significance was defined as p < 0.05. LOS, length of stay (measured in days); BMI, body mass index; TBSA, total body surface area (measured in percentage); SD, standard deviation; age (measured in years).

Variable	Overall (Mean ± SD)	p-value
LOS	3.1 ± 4.4	-
Age	6.9 ± 6.2	<0.001
BMI	21.4 ± 8.4	0.058
TBSA	5.5 ± 6.7	<0.001

Table 2 summarizes the demographic and clinical characteristics. In this cohort, 102 (44%) were females, and 130 (56%) were males, with no statistically significant difference in LOS observed between genders (p = 0.241). Flame burns were the most common source of injury (43%), followed by scalds (35%), and other causes (chemical/electric/radiation) comprising 22%. Patients with flame burns had significantly longer inpatient days (p = 0.039) compared to scald burns. Patients with scald injuries had shorter hospital stays by approximately -0.59 days compared to flame burns. Patients with burns from other causes (chemical/electrical/radiation) had longer hospital stays by approximately 0.70 days compared to flame burns. Inhalation injuries were diagnosed in 17 patients (7.3%). However, there was no statistically significant relationship between inhalation injuries and LOS in this population (p = 0.748). Patients with inhalation injuries had a shorter hospital stay on average (-0.95 days) compared to those without, but this difference was not statistically significant (95% CI: -5.92 to 4.02). TVDs were recorded for six patients (2.6%) who required mechanical ventilation. A moderate positive correlation was observed between TVD and LOS (Pearson correlation = 0.493, p = 0.320), but the small sample size precluded statistical significance.

**Table 2:** Summary of pediatric burn outcomes by key variables Data are presented as frequencies (percentages). Statistical significance was defined as p < 0.05.

Variable	Overall	p-value
Gender		0.241
Female	102 (44)	
Male	130 (56)	
Inhalation injury		0.748
Yes	17 (7.3)	
No	215 (92.7)	
Burn source		0.039
Flame	100 (43)	
Scald	81 (35)	
Other	51 (22)	

## Discussion

This study aimed to identify key demographic and clinical factors that contribute to prolonged hospital stays and overall worse outcomes among pediatric burn patients located at a rural Level 2 Trauma Center and BICU. Notably, age, burn severity, and the source of the burn were significantly associated with worse outcomes. Identifying these factors may prompt early stabilization, management, and timely referrals when necessary, improving patient outcomes, particularly in rural areas such as Appalachia.

The results of this finding reveal a significant association between age and length of hospital stay, as patients over the age of 6.9 are more likely to experience prolonged hospital LOS. This finding may be explained by adolescents having more risk-seeking behaviors combined with a lower risk perception. In addition, the management of the adolescent is different from the management of the child, and these variations in care may further contribute to differences in outcome. This result aligns with current research by Jordan et al., who concluded that adolescents sustain severe burns more frequently than any other pediatric age group and, therefore, experience the highest mortality rates [9]. Within Appalachia, a heavily industrialized region, young adolescents often have exposure to hazardous work environments that increase their risk of a severe burn. Tailored strategies, such as education on proper work safety, may serve to mitigate these risks within this population.

Increased burn severity, measured by TBSA, was found to have significantly longer hospital stays. It is well established in the literature that a higher TBSA is a predictor of prolonged hospital stays. In our study, the median TBSA was 5.5% (SD ± 6.7), lower than the cited thresholds in the literature. Currently, there is varying evidence in prior studies regarding the critical TBSA threshold necessitating referral to specialized centers. Kraft et al. stated that a TBSA of 60% warrants immediate referral to a tertiary care center [7], while Toma et al. reported that patients with a TBSA of 40% experienced higher mortality rates [5]. Moreover, Güney et al. stated that pediatric patients with TBSA over 47.5% with the presence of inhalation injury have a five times higher risk of mortality [10]. The discrepancies noted for the critical threshold of TBSA warrant region-specific guidelines on TBSA requiring referrals. Region-specific TBSA guidelines specific to the level of care required may improve patient outcomes in rural communities by helping providers recognize their capacity to deliver care and identify when to refer to specialized centers.

In our study, flame burns were the most common source of injury (43%), followed by scald burns (35%), and other types (chemical, electrical, or radiation) comprising 22%. Burn source analysis revealed that flame burns were associated with longer hospital stays compared to scald burns. This finding contradicts current literature suggesting that scald burns are the most common type of burn within the pediatric population and are typically associated with significantly lower mortality compared to flame burns [11,12]. The findings in our study may be due to the small sample size. Additionally, these findings may reflect the occupational and cultural patterns unique to Appalachia, where adolescents are often exposed to fires through industrial work and leisurely activity. These findings suggest the importance of implementing fire safety education within local communities and the workplace.

Although inhalation injuries are clinically significant, they did not significantly predict LOS in this cohort (p = 0.748). In contrast, Kraft et al. concluded that patients with an inhalation injury have a mortality rate of 23% [7]. Recent studies have reported a 41.5% mortality rate in patients with an inhalation injury compared to 7.2% in patients without an inhalation injury [13]. Inhalation injuries are especially dangerous due to their cascade of insults that increase an individual's susceptibility to acute respiratory distress and respiratory failure, leading to significant morbidity and mortality [13,14]. Further, inhalation injuries are also a leading cause of early deaths in burn victims, underscoring the importance of a timely diagnosis and initiation of treatment [13]. It is unclear why no statistical significance was identified in this study between inhalation injury and LOS; however, the small sample size is likely attributable. Therefore, future studies with larger sample sizes are needed to determine the association between inhalation injuries in the pediatric population and LOS.

In this study, BMI exhibited a borderline association with LOS, indicating potential interactions with burn severity and recovery duration (p = 0.058). There is limited literature regarding the role of BMI as a predictor of LOS in pediatric burn patients. However, Kraft et al. stated that patients with a BMI ≥ 85th percentile have an altered post-burn acute phase and impaired inflammatory response [15]. Despite these findings, no statistical differences in the incidence of sepsis, multi-organ failure, or mortality in pediatric burn patients were observed [15]. In adult burn patients, BMI does not play a significant role in patient outcomes [16]. Interestingly, Al-Dolaimy et al. found that overweight or obese patients have a mortality rate 0.19 times higher than those with normal BMI, although this finding was not statistically significant [17]. Appalachia has the highest rate of obesity in the country, and the potential relationship between BMI and post-burn recovery merits further investigation [18]. Observing this association could lead to more effective treatments tailored to patients in this setting.

This study provides an in-depth analysis of the key predictors of prolonged hospital stays for pediatric burn patients at a rural Level 2 Trauma Center and BICU. However, several limitations exist within the current study. These include a retrospective design and a small sample size. The small sample size limited the analysis of key factors such as inhalation injury and TVD on LOS, and therefore, its overgeneralization should be cautioned.

Future studies should include larger, multicentered data collection to improve generalizability. Further, investigating socioeconomic factors and the presence of secondary comorbidities within the pediatric population may provide a more comprehensive understanding of key factors associated with prolonged hospital stays. Prospective studies, including data from multiple rural trauma centers on standardized referral guides among rural populations, are warranted to understand if streamlining early treatment strategies improves outcomes.

## Conclusions

This study demonstrates that age, burn severity, and source of burn are significant predictors of prolonged hospital stay in pediatric burn patients. However, variables such as inhalation injury and BMI did not significantly predict hospital duration. Recognizing these key predictors in LOS allows providers to identify high-risk patients early and initiate timely management. Primarily, the findings from this study may be used to improve patient outcomes by serving as a manual for the development of preventative efforts for rural communities to mitigate occupational and environmental exposure. Further, these results may provide the foundation for the development of regional care recommendations and referral guidelines tailored to the needs of rural trauma centers.
